# Successful Outpatient Management of Children at a Secondary Care Hospital in Pakistan in a Dengue Fever Epidemic and Their Clinical Outcomes

**DOI:** 10.1155/2021/3296448

**Published:** 2021-11-02

**Authors:** Ammara Farooq, Brekhna Aurangzeb, Taimur Khalil Sheikh, Huma Bashir, Maryam Ghuncha, Tehmina Mustafa

**Affiliations:** ^1^Department of Pediatrics, Federal General Hospital, Islamabad, Pakistan; ^2^Department of Pediatrics, Pakistan Institute of Medical Sciences, Islamabad, Pakistan; ^3^Department of Pediatrics, Al Nafees Medical College and Hospital, Islamabad, Pakistan; ^4^Department of Pediatrics, Fazaia Medical College, Islamabad, Pakistan; ^5^Centre for International Health, Department of Global Public Health and Primary Care, University of Bergen, Bergen, Norway; ^6^Department of Thoracic Medicine, Haukeland University Hospital, Bergen, Norway

## Abstract

**Background:**

There is limited published literature on the feasibility of WHO 2009 guidelines for the management of dengue fever (DF) in Pakistani children. This study aimed to assess the outcome of children with DF who received outpatient treatment according to these guidelines during a DF epidemic.

**Method:**

This was a prospective cohort study conducted at Federal General Hospital, a secondary care hospital, Islamabad, Pakistan, from 1st August to 31st October 2019. Using WHO DF 2009 guidelines, children ≤13 years, diagnosed as confirmed DF (NS1 Ag +), were classified into the outpatient (DF) or the inpatient group (DF with warning signs or severe dengue (SD)). The inpatient group was admitted to the Pakistan Institute of Medical Sciences, a tertiary care hospital, and discharged on recovery. These children were followed for the primary outcome, i.e., recovery or hospitalization by day 14 of enrollment. Additionally, clinical and laboratory features (Hb, HCT, TLC, PLT, and ALT) of the patients in the outpatient who remained stable with those who progressed to inpatient care during follow-up were compared; also, time of recovery of blood counts was assessed.

**Results:**

Of 93 children with DF, 87 (93.5%) received outpatient care at enrollment. Of these, 6 (7.8%) deteriorated by day 7 and were admitted to inpatient care. SD was present in 6/93 (6.4%) patients at presentation and were admitted. All children showed signs of recovery until day 14. Male gender (*p*=0.049), lower normal mean platelet (*p*=0.02), and high mean hematocrit (*p*=0.001) were associated with disease progression.

**Conclusion:**

The majority of children with confirmed DF who received outpatient treatment according to WHO 2009 guidelines were successfully managed. Additionally, confirmed DF with warning signs or SD were admitted and recovered. Regular follow-ups according to the guidelines are pertinent. Thrombocytopenia and high HCT were associated with disease progression.

## 1. Introduction

Dengue fever (DF) is a mosquito born viral infection. There are estimated 100 to 400 million infections every year and half of the world population is at risk of DF [1]. Its incidence has increased eightfold in the recent decades [1]. The major burden of the disease (70%) is in Asia [1]. DF is a single disease with a wide spectrum of presentation ranging from asymptomatic sero-conversion to mild DF or severe DF, which may lead to shock, severe bleeding, and death [2, 3]. It is an acute, self-limiting disease in most instances but a significant number results in hospital admissions principally among children [2]. Without proper treatment, the case fatality rate in severe DF is more than 20% and with timely intervention, it can be reduced to <1% [1, 4].

In Pakistan, sporadic and epidemic cases of DF are reported [5, 6]. DF epidemics impose a substantial burden on health services and incur considerable economic, social, and personal costs [4–6]. The hospitalization rates of persons suspected of DF are high [6], as doctors tend to err on the side of caution and admit them for monitoring. This leads to higher bed occupancy rates, although the majority of DF cases are unlikely to require or benefit from medical care in hospital for their mild form of illness [7, 8]. On the other hand, there is clinical evolution of symptoms, and patients not requiring hospitalization on initial assessment may end up in critical stage of the disease, warranting admission to intensive care [2]. World Health Organization (WHO) has developed guidelines for diagnosis, treatment, prevention, and control of DF [2, 3]. A multicenter study from 18 countries has reported that this system of classification is very useful and user friendly [1] and precisely define the level of clinical care required by the patients [2]. These guidelines become even more important in outbreaks when the health system is already overwhelmed [2, 9].

There is limited published literature to evaluate the outpatient care of DF in children in terms of outcome and study risk factors for severe DF in Pakistan. Hence, we conducted this study with the aim to evaluate the outcome of children with DF who received outpatient care according to the WHO 2009 DF guidelines during a DF epidemic in a limited resource setting.

## 2. Methods

A prospective cohort study was conducted on pediatric patients presenting with signs and symptoms suggestive of DF at the paediatric outpatient (OPD) or emergency department of Federal General Hospital Islamabad from 1st August to 31st October 2019, during a dengue epidemic. It is a secondary care hospital with radiology and pathology departments for outpatient and inpatient services and caters densely populated suburbs of Islamabad. It was designated as one of the Dengue Management Centres during the dengue epidemic 2019 by the Ministry of National Health Services Regulation and Coordination, Pakistan. The hospital staff was trained and evaluated in the WHO 2009 guidelines at the start of the epidemic season. Written information was placed in the wards. A Pediatrician and General physician were designated in the hospital to monitor strict implementation of the guidelines and reporting to the National Dengue Cell. Duty doctors were periodically checked for strict adherence to the guidelines. Dengue NSI Antigen Kits (Imuomed, USA) were given to the hospital laboratory by the Government of Pakistan. NS1 Ag test was performed for all children who presented with acute febrile illness >48 hours and coming from the outbreak area.

Inclusion criteria were patients aged 1–13 years presenting with history of fever >48 hours with no obvious focus of infection and coming from the specified outbreak area, or history of fever >24 hours with headache, eye pain, joint pain, vomiting, bleeding, petechiae, or altered conscious level with positive NS1 antigen test for DF. Children having fever with an obvious focus of infection or other confirmed chronic diseases or those with negative NS1 antigen were excluded [3]. Patients fulfilling inclusion criteria were enrolled after informed consent from the caregiver or the parent. The duty doctors triaged the patients. They assessed patients for vital signs, evidence of bleeding, abdominal tenderness, hepatomegaly, and hydration status at initial presentation and on subsequent visits. Hemoglobin (Hb), hematocrit (HCT), total leukocyte count (TLC), total platelet count (PLT), alanine transferase (ALT), and NS1 antigen were checked in the laboratory of the hospital in all the patients fulfilling the inclusion criteria. The coagulation profile, chest X-ray, and abdominal ultrasound were done where indicated.

The children were classified based on clinical history, general and systemic examination, and laboratory tests as an outpatient group (DF) or an inpatient group (DF with warning signs and severe dengue) according to the WHO 2009 guidelines. The warning signs were abdominal pain or tenderness, persistent vomiting (unable to tolerate orally), not passing urine for 4 to 6 hours, evidence of mucosal bleed, petechiae on skin, hepatomegaly, evidence of clinical fluid accumulation (pedal edema, ascites, or pleural effusion), and increase in HCT with concurrent rapid decrease in PLT. Severe dengue was defined as one or more of these: severe bleeding, signs of shock (cold, clammy skin, prolong capillary refill, weak pulse, low blood pressure), signs of fluid accumulation (tender liver >2 cm or pedal edema or pleural effusion), signs of organ damage (liver; AST or ALT ≥1000, neurological and renal impairment) [3].

Hypotension was defined as narrow pulse pressure (<20 mm Hg) or hypotension for age (this is defined as systolic pressure <80 mmHg for those less than five years of age, or <90 mmHg for those five years of age and older). Fast breathing was defined in children >2–12 months as ≥50 breaths/min, 1 year to 5 years as ≥40 breaths/min, and >5 years as ≥30 breaths/min. High HCT was taken as ≥40%. TLC of less than 4000 × 10^3^/*μ*L was taken as leukopenia. Thrombocytopenia was defined as PLT of less than 150,000/mm^3^. Severe thrombocytopenia was defined as PLT of 50,000/mm^3^ or less. Percent change in hemoconcentration was taken as the difference between HCT maximum minus HCT minimum divided by HCT minimum multiplied by 100 [3].

The outpatients were given symptomatic treatment such as antipyretic, antiemetic, and oral rehydration [3]. The inpatient group was referred to the Pakistan Institute of Medical Sciences, Islamabad, Pakistan, a tertiary care government hospital, for admission.

### 2.1. Follow-Up

The patients were followed up on an outpatient basis until day 14 with symptoms, clinical examination to monitor for warning signs, clinical deterioration, or recovery according to the guidelines. The temperature recordings and blood counts (Hb, HCT, TLC, and PLT) and any other test as considered necessary by the treating physician were repeated daily for the first two follow-ups and then after 2 days interval till the improving trend occurred and patients recovered. The criteria for recovery were being afebrile for 48 hours, improvement in appetite, clinical well-being, and rising trends in PLT according to the standard protocol. If the patients defaulted to follow-up, they were traced on the telephone, and the reason for defaulting and their clinical symptoms were asked and recorded. The hospitalized patients in Pakistan Institute of Medical Sciences were followed up initially on phone and then in the outpatient clinic of Federal General Hospital Islamabad after they were discharged.

### 2.2. Outcome Measures

The primary outcome measure was defined as recovery or hospitalization until day 14 of enrollment of the children with DF treated on an outpatient basis. The secondary outcomes were (i) clinical features and biochemical parameters of children treated as outpatients and inpatients, (ii) comparison of outpatients who deteriorated after initial visits with those who continued as outpatients, and (iii) duration for the normalization of blood counts.

### 2.3. Sample Size

The sample size was calculated by WHO sample size calculator [10]. As the true hospitalization rate among those children with DF who received outpatient treatment in our local clinical settings was not available, therefore, we assumed a recommended 50% hospitalization rate [4]. Using 10% deviation and 95% confidence interval (CI), we required 96 children with DF.

### 2.4. Data Analysis

The data were entered and analyzed using Statistical Package for Social Sciences (SPSS) version 26. Descriptive statistics were performed to obtain frequency and percentages of categorical variables. Mean and standard deviation (SD) were reported for continuous variables. The Chi-square test and independent *T* test, where appropriate, were used for finding statistical significance among different variables. *P* value of <0.05 was considered statistically significant.

## 3. Results

**Figure 1:**
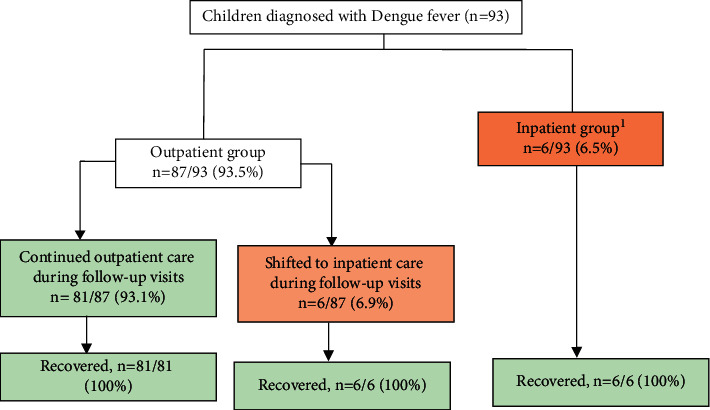
Flowchart of management and outcome of enrolled patients. Six patients were enrolled in the inpatient group on admission, and later on six patients from the outpatient group deteriorated and were shifted to the inpatient group. ^1^All these children had severe dengue, according to WHO criteria at presentation. Of these, 2 (33.3%) had severe bleeding with hypotension and 4 (66.7%) had signs of fluid accumulation and shock.

A total of 93 pediatric patients diagnosed with DF were enrolled in our study.  Figure 1 shows the flowchart of included patients, their place of management, and outcome. Of 93, 6 (6%) children had SD at the time of enrollment, and all of them were referred for inpatient treatment. In all, 87 (94%) patients were assigned to the outpatient group. Among these, 5 (6%) showed warning signs, while one (1%) had SD during the follow-up, and all of them were referred for inpatient treatment. In contrast, the remaining 81 (93%) recovered as outpatients. Of these, 10 (11%) children did not visit the clinic and were followed up for outcome by phone call and all recovered by day 14.

### 3.1. Baseline Characteristics of All Patients

The comparison of baseline demographic and clinical features of children with confirmed DF in the outpatient and inpatient groups is shown in Table 1. There was female predominance (54/93, 54.8%), and the mean age was 7.9 ± 2.8 years. Fever, body aches, and burning eyes were present in all the cases, followed by headache (45.2%) and vomiting (43%) as the most common symptoms. The proportion of children who presented with vomiting and diarrhea was significantly higher in the inpatient group compared to the outpatient group. There were no atypical clinical features such as splenomegaly, encephalitis, and refractory shock. Patients with severe DF had a higher mean age and longer duration of illness. The mean hospital stay of the inpatients was 9 ± 2 days. Duration of illness in SD was longer as compared to children with DF.

Table 2 shows the comparison of baseline laboratory investigations of the inpatient and outpatient groups. There was a marked difference in the means of TLC, PLT, and HCT in both the groups, which were statistically significant. ALT and AST were moderately raised in 50% of children in the inpatient group, whereas it was mildly raised in about 3.4% of children in the outpatient group. Pleural effusion and ascites were the notable imaging findings in the inpatient group.

### 3.2. Clinical and Biochemical Characteristics of Outpatients

**Figure 2:**
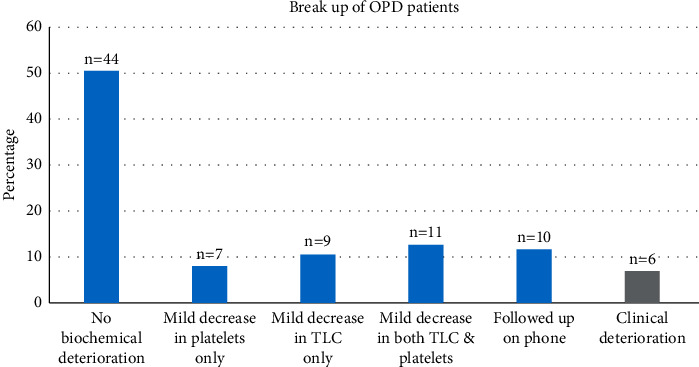
Clinical and biochemical characteristics of children with dengue fever who received outpatient treatment (*n* = 87). Despite mild biochemical abnormalities, no clinical deterioration occurred in 44/87 (50.5%) of patients. Five patients developed warning signs and one SD and were successfully picked.

Most patients were successfully treated and followed up on outpatient basis. There were no clinical deterioration or biochemical abnormalities in 44/87 (50.5%) patients (Figure 2). In others, mild TLC (*n* = 9/87, 10.3%) or PLT (*n* = 7/87, 8%) or both abnormalities (*n* = 11/87, 12.6%) were present. Though 27 (38%) patients developed mild fall in blood counts still there was no clinical deterioration. Six patients deteriorated after the initial visit and were successfully picked during follow-up visits and were shifted to inpatient.

### 3.3. Follow-Up of Children with Confirmed DF Who Received Outpatient Treatment

On follow-up visits, of 87 children with confirmed DF who received outpatient care, five patients developed warning signs, and one progressed to SD due to shock because of severe bleeding and were shifted to inpatient care. Gum bleed, epistaxis, and petechiae were the common bleeding manifestations (Table 3).

### 3.4. Comparison of Baseline Characteristics of the Patients Who Deteriorated versus Those Who Remained in the Same Group on Follow-Up

Table 4 shows the comparison of baseline characteristics of DF patients in the outpatient group who developed warnings signs (*n* = 5) or SD (*n* = 1) on follow-up and were shifted to the inpatient group as compared to the patients who remained and recovered in the outpatient group (*n* = 81). Male gender, fast breathing, high HCT, and lower limit of normal PLT count at baseline were associated with progression to severe disease. The mean of minimum and maximum HCT of the two groups was significantly different, whereas the change in percent hemoconcentration was 3.6% for the group who did not deteriorate on follow-up versus 4.9% for the group who deteriorated on follow-up. One child progressed to SD because of severe bleeding on follow-up and had a duration of fever for 7 days.

### 3.5. Shock in Dengue

Six patients had severe dengue with shock at initial presentation. Among them four had signs of fluid accumulation and shock, while two had severe bleeding with hypotension. The six patients who later deteriorated from the outpatient group, 1 developed shock due to bleeding. Male gender was a risk factor for shock. HCT was significantly higher in SD with shock.

### 3.6. Normalization of Leukopenia and Thrombocytopenia

**Figure 3:**
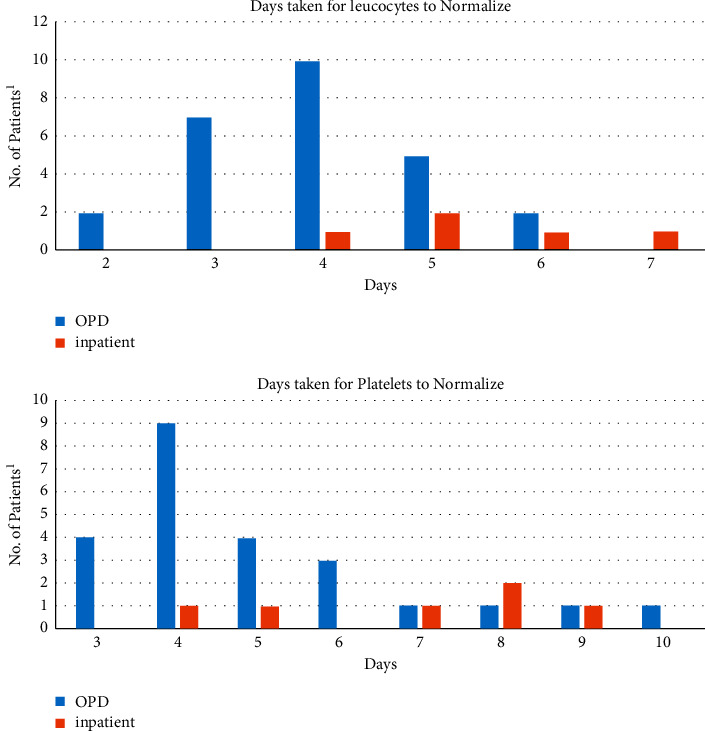
Time taken for the normalization of total leucocyte counts (TLC) and platelet counts (PLT) in patients who had deranged counts during the illness. PLT improved later than TLC. Maximum normalization occurred around 4 days after the time the values first deteriorated from normal. ^1^39/93 (41%) patients had deranged blood counts.

Figure 3 shows the pattern of normalization of TLC and PLT in all patients. The majority of patients with DF had normal values for TLC and PLT. Leucopenia and thrombocytopenia were most prevalent on day 6 of illness. In those who had deranged values, time taken for normalization ranged from 2 to 9 days with maximum normalization occurring four days after the time the values first deviated from normal.

## 4. Discussion

In this prospective study, the WHO 2009 DF management guidelines were implemented at a secondary and tertiary care health facility in Pakistan to assess the outcomes of children who received outpatient treatment. To our knowledge, to date, no published study has been found to evaluate the outcome of children who received outpatient treatment according to WHO 2009 DF guidelines in Pakistan. In this study, the majority of the children with DF received outpatient care with complete recovery within 14 days of enrollment. This shows that by following these guidelines and implementing them in a standardized way, a substantial majority of children with DF can be managed on outpatient basis with a positive outcome, thus reducing hospital inpatient burden. However, seven children with DF developed severe DF or DF with warning signs during the current episode of illness and were admitted for inpatient care and were successfully discharged due to timely and appropriate management at the tertiary care hospital. This finding also highlights that when appropriately applied, these guidelines help identify and manage disease deterioration. These findings are essential for clinicians working in our clinical settings and public health practitioners dealing with an epidemic of DF in Pakistan.

In our study, in total, 13% of children received inpatient care when we applied WHO criteria for admission. Similarly, using the same criteria for severe DF as given in the WHO guidelines, several studies have shown that severe DF ranged from 9% to 97% in their study population [2, 5, 9, 11]. Other studies from Singapore [10, 12] using modified criteria for inpatient treatment reported a higher proportion of admissions (one in four patients) during the epidemic of DF than what we have found. The range of severe DF that needs hospitalization depends on several factors such as the prevalent strains of dengue virus in the region, the catchment area (urban, rural) of the health center, environmental conditions of the area, and level of care of the health facility [13].

Another salient feature in our study was that seven children had SD with plasma leakage as the major cause who received inpatient treatment and were recovered. The median duration of fever among these children was five days, which corresponds to the critical period of disease. The clinical course of the disease is that the first phase is the febrile phase, which lasts for two to seven days. It is followed by the critical phase in which patient may go into shock either because of severe plasma leakage with high hematocrit or shock because of severe bleeding with low hematocrit, which lasts for 24 to 48 hours, and then the recovery phase, which occurs after 48 to 72 hours of the previous phase [14]. This study supports the findings of other researchers that three to eight days after onset of fever is a crucial period for monitoring patients with DF to have favorable outcomes [3, 14]. It also emphasizes that DF is a continuous rather than a distinct clinical entity, and it has an unpredictable clinical course; therefore, close monitoring in the critical period is essential [2, 3]. A prospective cohort study conducted on inpatients in Jakarta also revealed that the classification of the patients changed with following the cohort during the course of illness [11]. It also highlights that these guidelines are useful in identifying the different stages of the disease to make the appropriate decisions at the right time, as suggested by others [15].

Male gender, higher mean HCT, tachypnea, and lower normal PLT count at the time of enrollment were significantly associated with the progression of disease in our study. Male may be at risk for SD because they spend more time outside as compared to girls and are more at risk of mosquito bites and secondary infections, which increases the risk for SD [16]. This suggests that SD depends on the viral load and host factors such as secondary immune response [17]. However, a meta-analysis revealed no association of gender with severe DF [18]. Also, the patients who deteriorated later had mean TLC and PLT counts near the lower limit of normal at initial presentation compared to patients with DF who did not progress to SD. Similarly, high HCT and low PLT were reported to be significantly associated as risk factors in the febrile phase for progression to severe disease from recent systematic reviews and meta-analyses [17, 19] and other studies [20]. Tachypnea as a significant factor in these patients could be because of high grade fever or early sign of fluid accumulation in this group as compared to the other group [3]. Other studies have also shown that gastrointestinal symptoms such as vomiting/persistent vomiting, abdominal pain, tenderness, and gastrointestinal bleed at the time of presentation are associated with progression to SD [17, 18, 21]. Rash and thrombocytopenia were reported to be significant factors associated with SD and the disease progression by some researchers [22], but in our cohort, rash had almost the same distribution in both groups.

The monitoring of hematological parameters is of utmost importance in DF. The mean of maximum and minimum hematocrit percent between the children who deteriorated compared to those who did not deteriorate was significant in correspondence with the clinical course of the disease as there is rise in HCT as compared to baseline if the child has plasma leakage and fall or same HCT as compared to baseline HCT if the child has bleeding. However, the mean change in percent difference between the groups was not significant, which is probably because of the fact that in the deteriorated group only one child developed SD because of bleeding and others had DF with warning signs therefore there was not much percent difference to be expected between the two groups [3]. The little change in values may be the reason for less severe course of disease and fewer hospitalization in this cohort of children.

Regarding normalization of TLC and PLT, we found that PLT recovered later than TLC, with normalization in most children occurring four days after the time of deterioration. A study from Pakistan shows the same pattern [20]. This may be because there is transient bone marrow suppression both for leucopenia and thrombocytopenia. However, an additional cause for low PLT is antibody-mediated immunological destruction of PLT, as dengue antigens bind to PLT, which may be the reason for the delay in recovery of PLT compared to TLC [23, 24].

Although having strict admission criteria as defined by the WHO 2009 guidelines is beneficial to reduce hospital inpatient burden, adequate counseling of mothers/caregivers, frequent follow-up visits, and serial monitoring of clinical and hematological parameters of these children are of utmost importance to identify children with SD. Also, as shown in our study, inpatient management at a tertiary care hospital with adequate quality of care prevents poor outcomes in these children. Nevertheless, to implement these guidelines during the DF epidemic, a refresher training of clinical staff, appropriate logistic support, laboratory services including availability of the rapid diagnostic test to confirm DF (e.g., NS1Ag), timely referral to a tertiary care hospital with facilities for inpatient care, community advocacy, and awareness are required. Policymakers and district health departments should implement these guidelines at the beginning of the outbreak of DF. These data may help them make necessary arrangements, including the number of beds designated for SD patients and logistic supplies for their clinical settings. This study will also help our clinical staff to implement these guidelines in their clinical settings. The best outcome was possible because we had a tertiary care hospital back up for early referral, and the importance of which should not be overlooked.

This study has some strengths. First, it is a prospective study in which the patients were managed according to the WHO 2009 guidelines. Second, the current study was conducted at the dengue management center in a secondary care hospital in the capital city of Pakistan with an extensive catchment area. This center was designated as the dengue management center for the first time after training the health staff in these guidelines and provided services 24/7. Third, we validated the clinical skills of the health staff on a regular basis. Fourth, a good referral system was established for the inpatient management of children with SD at a tertiary care hospital. Fifth, appropriate sample size was used to evaluate the outcome of children with DF when they received outpatient treatment. At the start of the study, 50% hospitalization rate was assumed as true incidence in our area was not known. In our study, the hospitalization rate was 7% among those children with DF who received outpatient treatment. Using this true hospitalization rate (7%), with 5% deviation and 95% confidence interval, we needed 92 children with DF. Therefore, the reliability of the study is not affected. Last, we performed appropriate statistical analysis to examine the differences between the two treatment groups.

It is a single center study, which is one of the limitations. Therefore, it is important to conduct a multicenter study with an appropriate sample size and strict follow-up. Further, we could not follow 10 children with DF who received outpatient treatment in our clinic. There is a possibility that these children might have low hematological parameters during the follow-up period. However, we collected the information about the health status, including hospitalization during the follow-up period, through a telephone call by day 14 of the initiation of treatment. Moreover, the patients who developed warning signs but did not progress to shock might have been treated at our secondary care level setting if appropriate resources for a high dependency area could have been recruited. As the policy was for early referral and the required resources were not available at our end, we could not establish this finding, but it can be a step to be implemented in future for the validation of this idea.

## 5. Conclusion

The majority of children with confirmed DF who received outpatient treatment according to WHO 2009 guidelines were successfully managed on outpatient basis. In addition, children having confirmed DF with warning signs or SD were admitted and recovered. Vigilant initial assessment and robust regular follow-up with clinical signs and laboratory parameters according to the guidelines can successfully manage DF patients, both in inpatients and outpatients. Bleeding with thrombocytopenia and high hematocrit reflecting plasma leakage are associated with disease progression.

## Figures and Tables

**Table 1:** Comparison of baseline characteristics and clinical features of patients at initial presentation.

Characteristics	Total	Outpatient group^1^	Inpatient group^2^	*P* value
	(*n* = 93)	(*n* = 87)	(*n* = 6)	
Age (years)^3^, mean (±SD)	7.9 ± 2.8	7.8 (±2.8)	9.5 (±3.4)	0.154
Sex				
Male, *n* (%)	42 (45.2)	39 (44.8)	3 (66.7)	0.805
Female, *n* (%)	51 (54.8)	48 (55.2)	3 (33.3)	
Duration of fever at initial presentation (days), mean (±SD)	3.1 (±1.1)	3.0 (±0.9)	5.33 (±1.0)	0.000
Symptoms				
Fever, aches, pain, *n* (%)	93 (100.0)	87 (100.0)	6 (100.0)	-
Burning eyes, *n* (%)	93 (100.0)	87 (100.0)	6 (100.0)	-
Vomiting, *n* (%)	42 (45.2)	36 (41.4)	6 (100.0)	0.005
Headache, *n* (%)	40 (43.0)	36 (41.4)	4 (66.7)	0.226
Cough, *n* (%)	7 (7.5)	7 (8.0)	0 (0.0)	0.470
Diarrhea, *n* (%)	7 (7.5)	5 (5.7)	2 (33.3)	0.013
Backache, *n* (%)	5 (5.4)	5 (5.7)	0 (0.0)	0.546
Muscle soreness, *n* (%)	4 (4.3)	4 (4.6)	0 (0.0)	0.591
Gum bleed, *n* (%)	3 (3.2)	1 (1.1)	2 (33.3)	0.000
Abdominal pain, *n* (%)	3 (3.2)	1 (1.1)	2 (33.3)	0.000
Nosebleed, *n* (%)	2 (2.2)	0 (0.0)	2 (33.3)	0.000
Vitals				
Temperature (°F), mean (SD)	100.4 (±0.7)	100.4 (±0.7)	101.2 (±0.5)	0.008
Pulse (bpm), mean (SD)	106.0 (±5.6)	105.5 (±5.3)	113.7 (±5.0)	0.000
Respiratory rate				
Normal	85 (91.4%)	82 (94.2%)	3 (50.0%)	0.001
Fast breathing^3^	8 (8.6%)	5 (5.8%)	3 (50.0%)	
Blood pressure				
Normal	87 (93.5%)	87 (100%)	0 (0.0%)	0.000
Hypotension^4^	6 (6.5%)	0 (0.0%)	6 (100.0%)	
Signs				
Some dehydration, *n* (%)	39 (42.0)	34 (39.1)	5 (83.3)	0.034
Rash, *n* (%)	5 (5.4)	5 (5.7)	0 (0.0)	0.546
Red eyes, *n* (%)	9 (9.7)	8 (9.2)	1 (16.7)	0.549
Tender abdomen, *n* (%)	4 (4.3)	0 (0.0)	4 (66.7)	0.000
Hepatomegaly, *n* (%)	3 (3.2)	0 (0.0)	3 (50.0)	0.000
Ascites, *n* (%)	4 (4.3)	0 (0.0)	4 (66.7)	0.000
Petechiaem, *n* (%)	1 (1.1)	0 (0.0)	1 (16.7)	0.000
Pleural effusion, *n* (%)	1 (1.1)	0 (0.0)	1 (16.7)	0.000

^1^All these children had DF according to WHO criteria with NS1Ag confirmation. ^2^All these children had SD, according to WHO criteria at presentation with NS1Ag confirmation. Of these six children, 4 had shock due to severe plasma leakage and 2 had severe bleeding. The median duration of fever before development of SD was 5 days. Mean duration of hospital stay was 9 (±2) days. ^3^Fast breathing was defined as children 2–11 months = ≥50 breaths/min, 12–59 months = ≥40 breaths/min, and >5 yr = ≥30 breaths/min. ^4^Hypotension was defined as narrow pulse pressure <20 mm Hg. DF: dengue fever; bpm: beats per minute; SD: standard deviation.

**Table 2:** Comparison of baseline laboratory investigations of the outpatient and inpatient groups at initial presentation.

Investigations	Outpatient group (DF)^7^ (*n* = 87)	Inpatient group (severe DF)^7^ (*n* = 6)	*P* value
Hemoglobin (g/dl), mean ± SD	11.0 ± 1.1	11.3 ± 1.1	0.649
Total leucocyte count (x10^3^/*μ*l), mean ± SD	5235.6 ± 1218.9	3350 ± 665.6	0.020
Range of total leucocyte count (x10^3^/*μ*l)	2700–9500	2200–4700	—
Total leucocyte count <4,000 (x10^3^/*μ*l), *n* (%)	20 (23)	6 (100)	—
Total platelet count (x10^3^/*μ*l), mean ± SD	195.8 ± 43.4	65.2 ± 35.0	0.000
Range of platelets (x10^3^/*μ*l)	72,000–316,000	16,000–150,000	—
Platelet count <150,000 (x10^3^/*μ*l), *n* (%)	24 (28)	6 (100)	
Platelet count <50,000 (x10^3^/*μ*l), *n* (%)	0	6 (100)	—
Hematocrit (mean ± SD)	36.4 ± 1.8	43.3 ± 3.9	0.000
Range of hematocrit	33–42	39–48	

ALT raised, *n*/*N*^1^ (%)			
Mildly raised (40–75 IU/L)	3 (4.1)	0	0.000
Moderately raised (76 to 999 IU/L)	0	3 (50.0)	
Markedly raised (≥1000 IU/L)	0	0	

AST raised–*n*/*N*^2^ (%)			
Mildly raised (40–75 IU/L)	3 (4.1)	3 (50)	0.000
Moderately raised (76 to 999 IU/L)	0	0	
Markedly raised (≥1000 IU/L)	0	0	

CXR abnormality, *n*/*N*^3^ (%)	0	1^4^ (16.7)	0.000
USG abdomen abnormality, *n*/*N*^5^ (%)	0	2^6^ (33.3)	0.000

^1^ALT was done in 78 patients in total (72 in outpatient and 6 in inpatient). ^2^AST was done in 73 patients in total (67 in outpatient and 6 in inpatient), ^3^CXR was done in 10 children in total (5 in outpatient and 5 in inpatient). ^4^Pleural effusion. ^5^USG abdomen was done in 13 children in total (8 in outpatient and 5 in inpatient). ^6^Ascites. ^7^All these children had DF according to WHO criteria with NS1Ag confirmation. ^7^All these children had SD, according to WHO criteria at presentation with NS1Ag confirmation.

**Table 3:** Identification of admission criteria among six children with dengue fever managed on outpatient basis that showed signs of deterioration on follow-up^1^.

Parameters	Patient 1	Patient 2	Patient 3	Patient 4	Patient 5	Patient 6
Duration of fever at initial presentation (days)	3	3	2	3	3	2
Duration of fever when admitted (days)	6	7	7	6	7	6

Criteria for admission Dengue with warning signs						
Abdominal pain or tenderness		+	+	+		
Persistent vomiting						
Clinical signs of fluid accumulation						
Mucosal bleed	+	+	+	+	+	+
Lethargy, restlessness		+		+	+	
Liver enlargement >2 cm		+			+	
Laboratory: increase in hematocrit with concurrent rapid fall in platelets	+	−	+	+	+	+

Criteria for severe dengue						
Severe plasma leakage leading to: Shock (DSS) OR Fluid accumulation with respiratory distress Severe bleeding as evaluated by clinician Severe organ involvement Liver; AST, or ALT ≥1000 CNS; impaired consciousness Heart and other organs		+ + +				
Duration of hospitalization (days)	8	14	7	10	5	4

^1^Five children had warning signs, while one child had severe dengue according to WHO Criteria. AST: aspartate transaminase; ALT: alanine transaminase; CNS: central nervous system; DSS: dengue shock syndrome.

**Table 4:** Comparison of baseline characteristics of patients with dengue fever who remained stable with the patients who had deterioration of disease on follow-up.

	Dengue fever (*n* = 81)	Deterioration of disease (*n* = 6)	*P* value
Age (years) ± SD	7.65 ± 2.77	9.67 ± 2.50	0.087

*Sex*			
Male, *n* (%)	34 (42)	5 (83.3)	0.049
Female, *n* (%)	47 (58)	1 (16.7)	
Duration of illness in days (mean ± SD)	3 ± 0.95	2.67 ± 0.52	0.399

*Vitals*			
Pulse (bpm) (mean ± SD)	105.3 ± 5.27	108 ± 5.8	0.232
Temperature (°F) (mean ± SD)	100.3 ± 0.73	100.8 ± 0.41	0.096
Hypotension	0	0	—
Fast breathing	3 (6.2%)	2 (33.3%)	0.009

*Clinical features*			
Fever, aches, pain, burning eyes, *n* (%)	81 (100)	6 (100)	—
Vomiting, *n* (%)	33 (40.7)	3 (50)	0.657
Headache, *n* (%)	34 (42)	2 (33.3)	0.678
Cough, *n* (%)	6 (7.4)	1 (16.7)	0.421
Diarrhea, *n* (%)	5 (6.2)	0	0.531
Rash, *n* (%)	5 (6.2)	0	0.531
Abdominal pain, *n* (%)	1 (1.2)	0	0.784
Muscle soreness, *n* (%)	4 (4.9)	0	0.577
Petechiae, *n* (%)	0	^1^0	
*Investigations*			
Hemoglobin (g/dl) (mean ± SD)	11.04 ± 1.08	11.02 ± 1.08	0.952
Total leucocyte count (x10^3^/*μ*l) (mean ± SD)	5280.2 ± 1247.7	4633.3 ± 422.7	0.212
Range of total leucocyte count (×10^3^/*μ*l)	2800–9500	2700–5400	
Number of patients with total leucocyte count <4000 (×10^3^/*μ*l)	20 (24.7%)	6 (100%)	—
Total platelet count (×10^3^/*μ*l) (mean ± SD)	198.7 ± 43.4	156.8 ± 108.7	0.022
Range of platelets (×10^3^/*μ*l)	73–316	16–178	
Number of patients with platelet count <150000 (×10^3^/*μ*l)	18 (22%)	6 (100%)	
Number of patients with platelet count <50,000 (×10^3^/*μ*l)	0	5 (83.3%)	
Hematocrit (%) (mean ± SD)	36.3 ± 1.7	38.7 ± 1.3	0.001
Range of hematocrit (%)	33–41.5	38–46	
Hematocrit maximum (%) (mean ± SD)	37.6 ± 1.9	40.6 ± 2.9	0.001
Hematocrit minimum (%) (mean ± SD)	36.3 ± 1.7	38.7 ± 1.3	0.001
^6^% Hemoconcentration change (mean ± SD)	3.7 ± 1.8	4.9 ± 4.9	0.179
PT deranged mild	0	5 (83.3%)	
APTT	0	4 (66.7%)	
USG abdomen	0	^2^0	—
CXR	0	^3^0	—
^4^Raised ALT	2 (3.0%)	^7^2 (33.3%)	—
^5^Raised AST	2 (3.0%)	^7^2 (33.3%)	—

^1^Two patients later on developed petechiae. ^2^Later USG showed ascites in one and gall bladder wall odema in other patient. ^3^Later 1 patient showed pleural effusion. ^4^ALT was done in 72 patients in total (66 in DF and 6 in those who deteriorated). ^5^AST was done in 67 patients (61 in DF and 6 in those who deteriorated). Both groups had mildly raised ALT and AST at initial presentation. ^6^%Hemoconcentration change (HCT maximum–HCT minimum/HCT min x100). ^7^Two patients, one with previously normal liver enzymes and one with previously mildly raised enzymes, developed markedly raised enzymes later on.

## Data Availability

The underlying data supporting the results of the study can be made available from the corresponding author upon reasonable request.
